# Emergency air evacuation of patients with acute respiratory failure due to SARS-CoV-2 from Mayotte to Reunion Island

**DOI:** 10.1097/MD.0000000000027881

**Published:** 2021-12-03

**Authors:** Hamza Berguigua, Ludovic Iche, Philippe Roche, Cyril Aubert, Renaud Blondé, Antoine Legrand, Bérénice Puech, Chloé Combe, Charles Vidal, Margot Caron, Marie-Christine Jaffar-Bandjee, Christophe Caralp, Nora Oulehri, Hugo Kerambrun, Jérôme Allyn, Yvonnick Boué, Nicolas Allou

**Affiliations:** a Department of Emergency, Center Hospitalier Universitaire Felix Guyon, Saint Denis, France; b Department of Emergency, Center Hospitalier de Mayotte, Mamoudzou, France; c Réanimation Polyvalente, Center Hospitalier de Mayotte, Mamoudzou, France; d Réanimation polyvalente, Center Hospitalier Universitaire Felix Guyon Allée des Topazes Saint Denis, France; e Microbiologie, Centre Hospitalier Universitaire Felix Guyon Allée des Topazes Saint Denis, France; f Département d’Informatique Clinique, Centre Hospitalier Universitaire Felix Guyon Allée des Topazes, Saint Denis, France.

**Keywords:** air evacuation, COVID-19, Mayotte, Reunion Island, SARS-CoV-2, transportation

## Abstract

In February 2021, an explosion of cases of severe acute respiratory syndrome coronavirus 2 (SARS-CoV-2) pneumonia overwhelmed the only hospital in Mayotte. To report a case series of patients with acute respiratory failure (ARF) due to SARS-CoV-2 who were evacuated by air from Mayotte to Reunion Island.

This retrospective observational study evaluated all consecutive patients with ARF due to SARS-CoV-2 who were evacuated by air from Mayotte Hospital to the intensive care unit (ICU) of Félix Guyon University Hospital in Reunion Island between February 2, and March 5, 2021.

A total of 43 patients with SARS-CoV-2 pneumonia were evacuated by air, for a total flight time of 2 hours and a total travel time of 6 hours. Of these, 38 patients (88.4%) with a median age of 55 (46–65) years presented with ARF and were hospitalized in our ICU. Fifteen patients were screened for the SARS-CoV-2 501Y.V2 variant, all of whom tested positive. Thirteen patients (34.2%) developed an episode of severe hypoxemia during air transport, and the median paO_2_/FiO_2_ ratio was lower on ICU admission (140 [102–192] mmHg) than on departure (165 [150–200], *P* = .022). Factors associated with severe hypoxemia during air transport was lack of treatment with curare (*P* = .012) and lack of invasive mechanical ventilation (*P* = .003). Nine patients (23.7%) received veno-venous extracorporeal membrane oxygenation support in our ICU. Seven deaths (18.4%) occurred in hospital.

Emergency air evacuation of patients with ARF due to SARS-CoV-2 was associated with severe hypoxemia but remained feasible. In cases of ARF due to SARS-CoV-2 requiring emergency air evacuation, sedated patients receiving invasive mechanical ventilation and curare should be prioritized over nonintubated patients. It is noteworthy that patients with SARS-CoV-2 pneumonia related to the 501Y.V2 variant were very severe despite their young age.

## 1 Introduction

Mayotte (270,000 inhabitants) and Reunion Island (845,000 inhabitants) are 2 French overseas departments located in the Indian Ocean at a distance of 10,000 km from Paris. Until January 2021, Mayotte was relatively spared by the severe acute respiratory syndrome coronavirus 2 (SARS-CoV-2) pandemic. On January 6, 2021, the incidence of deaths caused by SARS-CoV-2 was 20.7 per 100,000 inhabitants on the island (compared to 116.4 per 100,000 inhabitants in metropolitan France).^[1,2]^ However, the incidence of infection with SARS-CoV-2 rose rapidly from 268 per 100,000 inhabitants in week 3 to >800 per 100,000 inhabitants in weeks 5 and 6, an increase that has been attributed to the SARS-CoV-2 501Y.V2 variant.^[3]^ In February 2021, the only hospital of the island, Mayotte Hospital,^[1]^ was overwhelmed by the explosion in the number of cases, as it is equipped with only 16 intensive care unit (ICU) beds for a population of 270,000 inhabitants. The medical infrastructure in Reunion Island meets European standards (P3 laboratories, 16 extracorporeal membrane oxygenation machines, coronary angiography, surgeons trained to perform all types of surgeries, among others).^[4]^ When Mayotte Hospital became overwhelmed with cases of SARS-CoV-2 pneumonia, several patients were transferred to Félix Guyon University Hospital in Reunion Island, in a context of solidarity among islands of the Indian Ocean region. All patients had to be evacuated by air due to the emergency situation. There is no transplantation center (except kidney) on the French overseas territories of Reunion Island and Mayotte which are located in the Indian Ocean. As a result, patients needing heart, lung or liver transplantation must be transferred to Paris, over a distance of 10,000 km. It is to be noted that medical teams in both Reunion Island and Mayotte have experience with emergency air evacuations, including for patients receiving extracorporeal membrane oxygenation support.^[5,6]^ It was necessary to urgently transfer patients with acute respiratory failure (ARF) due to SARS-CoV-2 from Mayotte to the only ICU in Indian Ocean equipped with many VV-ECMO supports. To date, few studies have examined the outcome of patients with SARS-CoV-2 pneumonia evacuated by air,^[7–9]^ and only a handful of them have focused on intensive care patients.^[10,11]^ Here, we report a case series of patients with ARF due to SARS-CoV-2 who were evacuated by air from Mayotte to Reunion Island.

## 2 Methods

### 2.1 Study design

This retrospective observational study evaluated all consecutive patients with ARF due to SARS-CoV-2 who were evacuated by air from Mayotte Hospital in Mamoudzou to the ICU of Félix Guyon University Hospital in Reunion Island between February 2 and March 5, 2021. All patients or their legally authorized representative were verbally informed about the study and could refuse to participate; moreover, they received a written information notice about the process of data collection. The study was approved by the Ethics Committee of the French Society of Infectious Disease and Tropical Medicine (CER-MIT), and was declared to the *Commission nationale de l’informatique et des libertés* (French Data Protection Agency MR004, # 2206739). This study complies with the Strengthening the Reporting of Observational studies in Epidemiology recommendations statement.^[12]^

All evaluated patients had positive confirmatory respiratory sample with SARS-CoV-2 by real time reverse transcription polymerase chain reaction using kit targeting IP2 and IP4 regions and N gene. Samples from nasopharyngeal swabs or patient's respiratory specimens were extracted using NucliSens easyMAG system (BioMérieux). The genomes were secondly obtained by sequencing using the Oxford Nanopore technology and the Artic Network's overlapping amplicon protocol.^[13,14]^

### 2.2 Therapeutic management

In accordance with our protocol, all patients with ARF due to SARS-CoV-2 were treated with: a 3^rd^ generation cephalosporin or piperacillin-tazobactam after realization of respiratory sample; dexamethasone at a dosage of 6 mg/day for 10 days; systematic deworming with ivermectine or albendazole; enhanced systematic anticoagulation according to the guidelines of the French Society of Hemostasis and the French Society of Anesthesia and Intensive Care.^[15]^

High flow nasal cannula oxygenation was initiated in patients who need standard oxygen ≥9 L/min to maintain peripheral arterial oxygenation saturation ≥92%. The decision of the timing of intubation and mechanical ventilation was not protocolized but determined by the ICU team.

### 2.3 Details of air evacuation

The protocol of air evacuation had been established by the prehospital emergency medical system of La Reunion and Mayotte and there was no specific training for these transports. In order to be transported, patients had to meet the following criteria: FiO_2_ <0.6, PEEP <15, and norepinephrine <1 mg/h. The use of curares in patients receiving invasive mechanical ventilation was not protocolized and was left to the discretion of the medical team.

**Figure 1:**
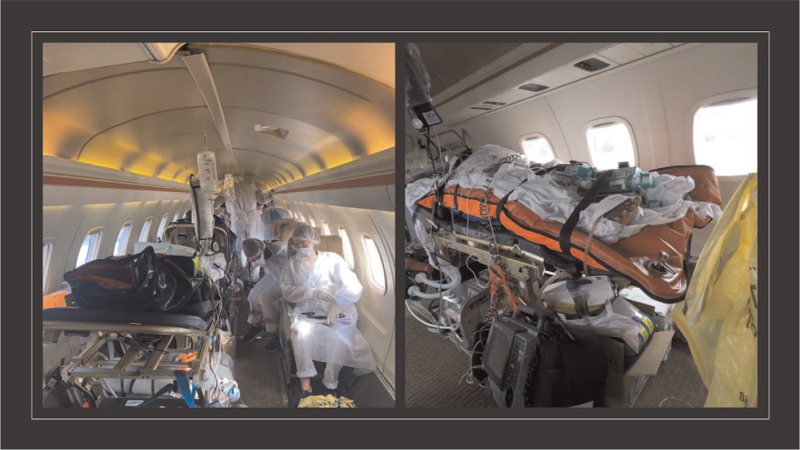
Air evacuation of patients.

Total flight time was 2 hours and total travel time between the 2 hospitals was 6 hours. Patients were evacuated on board an Embraer 135 aircraft specially equipped for medical evacuation missions (12 seats and 3 stretchers). The vast majority of flights carried 3 patients, 2 of whom received invasive mechanical ventilation and 1 received conventional oxygen therapy. The medical crew, was composed of 3 doctors and 3 nurses, experienced in inter-hospital transport. Both the cockpit and a delimited area at the bottom of the aircraft were considered “clean”; the rest of the aircraft was deemed “contaminated.” The medical team was fully dressed in protective clothing for the duration of the flight including examination gloves, filtering facepiece class 2 mask (FFP2) and goggles for eye protection. Each patient was assigned 1 doctor, 1 nurse, and 1 emergency kit composed of a vacuum mattress, an aspirator, a defibrillator, a transport ventilator, and suitcases with ventilation material and emergency medication (Fig. 1).

As there was no patient isolation unit and additional decontamination against SARS-CoV-2 was required between air transport.

### 2.4 Data collection

Clinical and biological data along with information on comorbidities were collected on admission to ICU.

Complications occurring during air transport and in ICU were recorded.

Survival at hospital discharge was also recorded

### 2.5 Outcomes

The primary outcome was the occurrence of severe hypoxemia during air transport (ie, oxygen saturation (spO_2_) <88% during >15 minutes).

The secondary outcomes were the need for veno-venous extracorporeal membrane oxygenation (VV-ECMO), duration of invasive mechanical ventilation, in-ICU length of stay and hospital mortality.

Other complications during air transport were evaluated: oxygen desaturation requiring increase of oxygen requirements or introduction of curares, hypotension (mean arterial pressure <65 mmHg) requiring positive inotropic therapy or fluid challenge >500 mL of crystalloid, endotracheal tube or vascular catheter dislodgement, arrhythmia, cardiac arrest, agitation requiring modification of sedation.

### 2.6 Statistical analysis

Categorical variables were expressed as total number (percentage) and continuous variables as median (25^th^–75^th^ percentiles). Categorical variables were compared using Fisher exact test. Continuous variables were compared using the nonparametric Wilcoxon test or Mann–Whitney *U* test, as appropriate. A *P* value <.05 was considered significant. Statistical analyses were conducted using SPSS 15.0 (SPSS Inc, Chicago, IL).

## 3 Results

Over the study period, 43 patients with SARS-CoV-2 pneumonia were evacuated by air from Mayotte Hospital in Mamoudzou to Félix Guyon University Hospital in Reunion Island. Of these, 38 patients (84.4%) presenting with ARF were transferred directly to our ICU and included in the study cohort. During air transport, 4 patients (10.5%) received conventional oxygen therapy (between 6 L/min and 9 L/min) before departure and 34 patients (89.5%) received invasive mechanical ventilation; 3 (8.8%) of the ventilated patients also received VV-ECMO support.

For all patients the indication for emergency air evacuation was to free up ICU beds in the hospital of Mayotte.

The characteristics of patients are shown in Tables 1 and 2. The median age of patients was 55 (46–65) years. Of the 38 evacuated patients, 44.7% had arterial hypertension, 36.8% were diabetic, and 34.2% had a body mass index >30 kg/m^2^. Fifteen patients (39.5%) were screened for the SARS-CoV-2 501Y.V2 variant, all of whom tested positive.

**Table 1:** Characteristics at hospital departure of the 38 patients with acute respiratory failure due to SARS-CoV-2.

	Total	Severe hypoxemia		
Characteristics	(n = 38)	Yes (n = 13)	No (n = 25)	*P*
Delay between diagnosis and onset of symptoms, days	6 (4–7)	7 (3–7)	6 (4–7)	.309
Delay between in ICU admission and onset of symptoms, days	7 (5–9)	7 (6–11)	7 (5–9)	.178
Delay between air transfer and onset of symptoms, days	12 (9–14)	12 (9–15)	13 (7–14)	.987
Delay between orotracheal intubation and onset of symptoms, days	4 (2–7)	5 (2–11)	4 (2–6)	.984
Comorbidities				
Age, y	55 (46–65)	50 (41–66)	56 (51–64)	.199
Male sex	29 (76.3)	10 (76.9)	19 (76)	.64
Chronic obstructive pulmonary disease	7 (18.4)	2 (15.4)	5 (20)	.549
History of congestive heart failure	4 (10.5)	0	4 (16)	.278
Chronic kidney disease	3 (7.9)	0	3 (12)	.538
Immunodepression	3 (7.9)	0	3 (12)	.538
Hypertension	17 (44.7)	4 (30.8)	13 (52)	.307
Body mass index >30 kg/m^2^	13 (34.2)	2 (15.4)	11 (44)	.148
Diabetes mellitus	14 (36.8)	3 (23.1)	11 (44)	.294
Organs failure the day of air transport				
Sequential Organ Failure Assessment score	5 (2–7)	3 (2–6)	5 (3–5)	.118
d-Dimer level, μg/mL	1248 (711–3404)	1210 (572–2270)	1395 (915–5613)	.303
Lactate dehydrogenase, IU/L	513 (477–679)	529 (480–685)	509 (468–621)	.734
Fibrinogen, g/L	6.69 (4.96–8.04)	7.4 (4.8–8.4)	6.7 (5.3–8)	.172
Lymphocytes count, /L	0.67 (0.47–0.99)	0.92 (0.58–1.1)	0.59 (0.46–0.84)	.249
Polynuclear neutrophils, /L	10.8 (8.45–14.3)	9.83 (6.09–13.2)	10.9 (9.5–14.6)	.109
C-reactive protein, mg/dL	101.4 (53–162)	162 (77–273)	84 (29–113)	.126
Creatinin, μmol/L	75 (57–119)	65 (55–89)	87 (58–125)	.2
Total bilirubin level, mg/dL	13 (9–18)	14 (11–20)	12 (8–18)	.46
Prothrombin time (%)	71 (64–76)	71 (67–79)	71 (63–76)	.564
Platelet count, G/L	213 (157–315)	192 (156–378)	221 (157–281)	.903
PaO_2_/FiO_2_ ratio	165 (150–200)	180 (83–202)	165 (150–200)	.94
Co/superinfection	6 (15.8)	1 (7.7)	5 (20)	.643
Glasgow Coma Scale score	15 (15–15)	15 (15–15)	15 (15–15)	.856
Invasive Mechanical ventilation	34 (89.5)	8 (61.5)	26 (100)	.003
Catecholamines	8 (21.1)	3 (23.1)	5 (20)	.568
Venoveinous extracorporeal membrane oxygenation	3 (7.9)	1 (7.7)	2 (8)	.99
Neuromuscular blocking agents	32 (84.2)	8 (61.5)	24 (96)	.012

**Table 2:** Characteristics at hospital admission (Reunion Island) of the 38 patients with acute respiratory failure due to SARS-CoV-2.

	Total	Severe hypoxemia		
Characteristics	(n = 38)	Yes (n = 13)	No (n = 25)	*P*
Respiratory parameters at hospital admission				
pH	7.42 (7.34–7.46)	7.45 (7.39–7.50)	7.36 (7.30–7.44)	.014
PaO_2_/FiO_2_ ratio	140 (102–192)	101 (63–112)	155 (124–199)	.007
paCO_2_ admission, mmHg	48 (42–58)	45 (37–60)	49 (44–58)	.447
Lactate, mmol/L	1.3 (1.1–1.6)	1.5 (1.3–2)	1.2 (1–1.3)	.006
Respiratory rate (/min)	25 (22–28)	26 (22–30)	25 (23–28)	.481
Positive end-expiratory pressure, cmH_2_O	12 (10–13)	13 (10–15)	11 (9–12)	.156
Plateau pressure, cmH_2_O	24 (21–26)	25 (21–32)	24 (21–25)	.444
Driving pressure, cmH_2_O	14 (10–16)	14 (9–24)	14 (10–16)	.868
Pulmonary infiltrates >50% extension on chest CT scan	25 (65.8)	9 (69.2)	16 (64)	.52
PaO_2_/FiO_2_ ratio on day 1 following admission	155 (104–205)	115 (68–202)	168 (134–220)	.084
Corticosteroid	38 (100)	13 (100)	25 (100)	.99
Enhanced anticoagaulation therapy	38 (100)	13 (100)	25 (100)	.99
Pulmonary infiltrates >50% extension on chest CT scan	25 (65.8)	9 (69.2)	16 (64)	.52

The median time from onset of symptoms to air evacuation was 12 (9–14) days. For patients receiving invasive mechanical ventilation, the median time from orotracheal intubation to air evacuation was 4 (2–7) days. Eight patients (21.1%) were treated with noradrenaline during air transport.

A total of 13 patients (34.2%) developed an episode of severe hypoxemia during air transport.

Delay between air transfer or delay between orotracheal intubation in mechanically ventilated patients and onset of symptoms were not associated with severe hypoxemia during air transport (*P* > .9) (Table 1). In univariate analysis, the only factors predicting the occurrence of severe hypoxemia during air transport was lack of treatment with curare (*P* = .012), lack of invasive mechanical ventilation (*P* = .003) (Table 1).

The median paO_2_/FiO_2_ ratio was significantly lower on admission to our ICU (140 [102–192] mmHg) than on departure from Mayotte Hospital (165 [150–200] mmHg, *P* = .022) (Table 2).

Three patients developed hypotension requiring positive inotropic therapy (mean arterial pressure <65 mmHg) and fluid challenge >500 mL of crystalloid during air transport.

Agitation requiring modification of sedation occurred in 2 patients (5.3%) and oxygen desaturation requiring increase of fraction of inspired oxygen, sedation enhancement and introduction of neuromuscular blocking agents occurred in 8 patients (21%).

No cardiac arrest, arrhythmia, endotracheal tube or vascular catheter dislodgement occurred during air transport.

The median plasma lactate concentration on admission to ICU was 1.3 (1.1–1.6) mmol/L (Table 2).

### 3.1 Evolution in ICU

One patient had a fatal cardiac arrest due to pulmonary embolism in our ICU within 24 hours of departure from Mayotte Hospital.

One patient who presented with acute coronary syndrome in Mayotte underwent coronary angiography in our ICU. The intervention revealed severe 3-vessel disease requiring revascularization.

Of the 35 patients who were not on VV-ECMO support on departure from Mayotte, 6 (17.1%) required VV-ECMO support for refractory acute respiratory distress syndrome in our ICU. Overall, 9 (23.7%) of 38 patients received VV-ECMO support for refractory acute respiratory distress syndrome (Table 3).

**Table 3:** Evolution during the stay in intensive care unit.

	Total	Severe hypoxemia		
Characteristics	(n = 38)	Yes (n = 13)	No (n = 25)	*P*
Renal replacement therapy	6 (15.8)	2 (15.4)	4 (16)	.672
Catecholamines	18 (47.4)	5 (38.5)	13 (52)	.506
Invasive mechanical ventilation	34 (89.5)	8 (61.5)	26 (100)	.003
Duration of invasive mechanical ventilation, days	20 (10–35)	31 (15–44)	19 (8–28)	.101
Prone position	28 (73.7)	8 (61.5)	20 (80)	.55
Prone positioning (sessions)	2 (1–4)	5 (2–7)	2 (1–4)	.012
Inhaled nitric oxide	9 (23.7)	3 (23.1)	6 (24)	.66
Extracorporeal membrane oxygenation	8 (21.1)	3 (23.1)	5 (20)	.568
Enhanced anticoagaulation therapy	38 (100)	13 (100)	25 (100)	.99
Duration of invasive mechanical ventilation, days	20 (10–35)	31 (15–44)	19 (8–28)	.101
Hospital-acquired pneumonia	19 (50)	5 (38.5)	14 (56)	.495
Pulmonary embolism	8 (21.1)	2 (15.4)	6 (24)	.282

Pulmonary embolism and hospital-acquired pneumonia occurred in 8 patients (21.1%) and 19 patients (50%), respectively.

The median length of stay in ICU was 21 (13–46) days, with no significant difference between patients with and without an episode of severe hypoxemia during air transport (*P* = .584) (Table 3).

After a median follow-up of 56 (49–66) days, 7 deaths (18.4%) occurred, with no significant difference between patients with and without an episode of severe hypoxemia during air transport (*P* = .385) (Table 3).

## 4 Discussion

In our study, 34.2% of patients with ARF due to SARS-CoV-2 developed an episode of severe hypoxemia during air transport. The respiratory status of patients deteriorated considerably between departure and arrival, as attested by the significant decrease in the median paO_2_/FiO_2_ ratio. The only factors significantly associated with severe hypoxemia during air transport were lack of treatment with curare and lack of invasive mechanical ventilation.

At the beginning of the SARS-CoV-2 pandemic, international guidelines recommended the early intubation of infected patients with respiratory distress ^[16]^ because noninvasive ventilation was believed to cause secondary contamination of caregivers via aerosolization.^[17]^ This risk has since been shown to be limited, and noninvasive ventilation is now recommended over invasive ventilation.^[18,19]^ However, in our study, almost all patients were intubated in Mayotte before being evacuated, as noninvasive mechanical ventilation proved ineffective due the severity of their condition. Note that this procedure was in accordance with international guidelines for air evacuation, which recommend stabilizing patients at risk of respiratory deterioration through preventive orotracheal intubation, whether or not they are infected with SARS-CoV-2.^[20]^

Studies have shown that the transfer of critically ill patients receiving mechanical ventilation can increase ventilation duration and patient mortality.^[21,22]^ However, the long-distance transfer of patients with ARF appears to be safe when performed by specialized and dedicated medical teams.^[23]^ In the study by Pavain et al, the inter-hospital transfer of patients with SARS-CoV-2 pneumonia receiving mechanical ventilation was not associated with an increase in mortality.^[10]^ In our study, no excess mortality was found in patients who developed an episode of severe hypoxemia during air transport, even though a significant deterioration of patients’ respiratory status was observed on arrival. In our practice, the use of curares was not protocolized and was left to the discretion of the medical team. Nevertheless, lack of treatment with curare was associated with severe hypoxemia during air transport. From a medical perspective, several patients in our study received a personal benefit from air evacuation. Thus, 9 patients with refractory acute respiratory distress syndrome (23.7%) were able to receive VV-ECMO support in Reunion Island, where 16 machines are presently available compared to 2 machines in Mayotte. Moreover, 1 patient who presented with acute coronary syndrome in Mayotte was able to undergo coronary angiography in Reunion Island; the intervention revealed three-vessel disease, prompting treatment with coronary revascularization. From an ethical perspective; however, the benefits of emergency air evacuation existed only at the collective level (eg, reduction in the need for triage).^[10,11]^ The lack of ethical benefit at the personal level explains why many patients and/or their families refused the evacuation.^[24]^ Lastly, it should be stressed that patients in our case series were critically ill, with almost a quarter of them requiring VV-ECMO support despite their young age. This could due to the increased virulence of variant of concern, which has been highlighted in several studies.^[25,26]^

The other issues to be highlighted are the numerous transfers to manage to reach the ICU at the hospital of Reunion Island (ICU to ambulance, ambulance to airplane…) and the discomfort of the protective equipment in a tropical zone where temperature and hygrometry are very high at that season which corresponded to austral summer.

Our study has many limitations. Biases may have been introduced due to the retrospective nature of the study. In addition, the number of evaluated patients was relatively small. Yet, in their study of patients with SARS-CoV-2 pneumonia evacuated by air, Pavain et al^[10]^ examined only 13 patients, none of whom were on VV-ECMO support; moreover, travel time was shorter than in our study. Similarly, the study by Turc et al^[11]^ evaluated only 36 patients (with mechanical ventilation but without VV-ECMO support) who traveled for a much shorter period of time (70 minutes) than in our study.

In conclusion, emergency air evacuation of patients with ARF due to SARS-CoV-2 501Y.V2 variant was associated with respiratory complications but remained feasible. In cases of ARF due to SARS-CoV-2 requiring emergency air evacuation, sedated patients receiving invasive mechanical ventilation and curare should be prioritized over non-intubated patients. It is noteworthy that patients with SARS-CoV-2 pneumonia related to the 501Y.V2 variant were very severe despite their young age.

## Author contributions

HB, YB, NA had full access to all the study data and take responsibility for the completeness of the data and the accuracy of the analysis.

*Study concept and design:* HB, IL, PR, CA, RB, AL, BP, CC, CV, MC, MCJB, CC, NO, HC, JA, YB, NA, LD

Acquisition of data: HB, YE, HK

*Analysis and interpretation of data:* HB, IL, PR, CA, RB, AL, BP, CC, CV, MC, MCJB, CC, NO, HC, JA, YB, NA, LD

Drafting of the manuscript and critical revision of the manuscript for important intellectual content: HB, IL, PR, CA, RB, AL, BP, CC, CV, MC, MCJB, CC, NO, HC, JA, YB, NA

Statistical analysis: NA, JA

*Funding:* Support was provided solely by institutional and/or departmental sources.

Administrative, technical and material support and study supervision: HB, IL, PR, CA, RB, AL, BP, CC, CV, MC, MCJB, CC, NO, HC, JA, YB, NA

**Conceptualization:** Hamza Berguigua, Ludovic Iche, Cyril Aubert, Renaud Blondé, Antoine Legrand, Bérénice Puech, Chloé Combe, Charles Vidal, Margot Caron, Marie-Christine Jaffar-Bandjee, Christophe Caralp, Nora Oulehri, Yvonnick Boué, Nicolas Allou.

**Data curation:** Hamza Berguigua, Ludovic Iche, Philippe Roche, Cyril Aubert, Renaud Blondé, Chloé Combe, Charles Vidal, Nora Oulehri, Hugo Kerambrun, Yvonnick Boué.

**Formal analysis:** Hamza Berguigua, Ludovic Iche, Cyril Aubert, Renaud Blondé, Antoine Legrand, Bérénice Puech, Chloé Combe, Charles Vidal, Christophe Caralp, Jérôme Allyn, Yvonnick Boué, Nicolas Allou.

**Investigation:** Hamza Berguigua, Philippe Roche, Renaud Blondé, Antoine Legrand, Bérénice Puech, Charles Vidal, Margot Caron, Marie-Christine Jaffar-Bandjee, Nora Oulehri, Hugo Kerambrun, Yvonnick Boué.

**Methodology:** Hamza Berguigua, Ludovic Iche, Renaud Blondé, Charles Vidal, Christophe Caralp, Nora Oulehri, Jérôme Allyn, Yvonnick Boué, Nicolas Allou.

**Project administration:** Hamza Berguigua.

**Resources:** Christophe Caralp, Nora Oulehri, Hugo Kerambrun.

**Supervision:** Hamza Berguigua, Ludovic Iche, Philippe Roche, Margot Caron, Hugo Kerambrun, Yvonnick Boué, Nicolas Allou.

**Validation:** Hamza Berguigua, Ludovic Iche, Jérôme Allyn, Yvonnick Boué, Nicolas Allou.
